# Treatment of Anaerobic Digester Effluent Using *Acorus calamus*: Effects on Plant Growth and Tissue Composition

**DOI:** 10.3390/plants7020036

**Published:** 2018-04-20

**Authors:** Tararag Pincam, Hans Brix, Arunothai Jampeetong

**Affiliations:** 1 Environmental Science Program, Faculty of Science, Chiang Mai University, Meuang, Chiang Mai 50202, Thailand; ptararg@gmail.com; 2 Graduate School, Chiang Mai University, Meuang, Chiang Mai 50202, Thailand; 3 Department of Bioscience, Aarhus University, Ole Worms Allé 1, 8000 Aarhus, Denmark; hans.brix@bios.au.dk; 4 Department of Biology, Faculty of Science, Chiang Mai University, Meuang, Chiang Mai 50202, Thailand

**Keywords:** *Acorus calamus*, nutrient removal, sweet flag, swine wastewater

## Abstract

The responses of *Acorus calamus* under greenhouse conditions for 56 days when exposed to three dilutions (25%, 50%, and undiluted) of anaerobic digester effluent from a swine farm were determined. Plant growth, morphology, pigments, and minerals in plant tissues as well as water quality were investigated. The plants grew well in all concentrations of anaerobic digester effluent with no statistically significant effects on plant growth and morphology, and without any toxicity symptoms. The NH_4_^+^ concentrations in leaves and roots and the NO_3_^−^ concentrations in leaves as well as the nitrogen, phosphorus, and potassium concentrations in the plant tissues increased with increasing effluent concentration. The nutrients in the anaerobic digester effluent were removed effectively (NH_4_-N > 99% removal; PO_4_-P > 80% removal), with highest removal rates in the undiluted digester effluent. The removal of total suspended solids (>80% in 42 days) and chemical oxygen demand (37–53%) were lower. The dissolved oxygen concentration in the anaerobic digester effluent increased overtime, probably because of root oxygen release. It is concluded that *Acorus calamus* could be a promising species for treating high-strength wastewater with high nutrient concentrations, such as effluents from anaerobic digesters as well as other types of agricultural wastewaters.

## 1. Introduction

Discharge of contaminated water from animal farms is a main non-point source of pollution affecting organisms in aquatic ecosystems, and potentially human health [1,2]. For decades, intensive livestock production has increased greatly due to the rising demand for livestock products [3]. High volumes of livestock wastewater are consequently produced and appropriate animal waste management is required. Anaerobic digestion is a viable technology for treating several types of organic waste such as municipal, agricultural, industrial, and animal waste. Anaerobic digestion can both produce bioenergy in the form of methane and also clean the wastewater [4,5]. However, effluent from anaerobic digester treating e.g., waste from swine farms still has high concentrations of nutrients particular nitrogen (N) and phosphorus (P) [6,7,8,9]. In the anaerobic digester, the nitrogenous matter is transformed to ammonium nitrogen (NH_4_-N) by biodegradation processes [10], and the organic phosphorus or polyphosphate is transformed to orthophosphate (PO_4_-P) by biological reduction or assimilation of microorganisms, resulting in high concentrations of NH_4_-N and PO_4_-P in the anaerobic digester effluent [11]. Furthermore, the dissolved oxygen level in the anaerobic digester effluent is very low due to the anaerobic processes. The high concentration of nutrients and low oxygen availability in the anaerobic digester effluent can affect the aquatic organisms in receiving natural waters, leading to biodiversity degradation. Therefore, further treatment of the effluent from anaerobic digesters is needed to protect the aquatic environment.

Constructed wetlands are an efficient, cost-effective, and eco-friendly biological technology for wastewater treatment [12]. Besides system designs, types of substrate and microorganisms that influence the treatment efficiency, plants also play important roles for the treatment processes e.g., by increasing sediment deposition, removing excessive nutrients and release of oxygen into the water and rhizosphere [13]. Several studies have documented that constructed wetlands with plants have higher treatment efficiency than unplanted systems. A study by Karathanasis et al. [14] found that constructed wetlands planted with *Typha latifolia* L. and *Festuca arundinacea* Schreb. had higher removal efficiency for Biochemical Oxygen Demand (BOD) and Total Suspended Solids (TSS) than unplanted systems. Likewise, the vertical subsurface flow constructed wetlands planted with *Acorus calamus* L. and *Lythrum salicaria* L. had high removal efficiencies for Chemical Oxygen Demand (COD), Total Nitrogen (TN) and Total Phosphorus (TP), compared with unplanted wetlands [15]. Most studies suggest that nutrient removal, particular N and P removal, was a result of plant uptake as plants need nutrients to support their growth. However, plants may suffer from stress conditions in high-strength wastewater, making their growth stunted and consequently their nutrient uptake low. Under excess nutrients and low oxygen in the anaerobic digester effluent from swine farm, plants performance is not well understood and it may negatively affect the treatment efficiency. Therefore, assessment of the effects of anaerobic digester effluent on plant growth is needed in order to evaluate the real potential of the plants to remove nutrients and improve water quality.

Sweet flag (*Acorus calamus*) is a herbaceous perennial wetland plant belonging to family Acoraceae. It is native to South Asia (particularly India), Central Asia, and Eastern Europe and it has distributed widely in the temperate and sub-temperate regions [16,17,18]. *Acorus calamus* grows rapidly especially in waterlogged and nutrient-rich soils [19,20,21] which makes it an obvious candidate species for use in the wastewater treatment systems. A previous study found that *A. calamus* had potential to remove COD (61–66%), TN (41–63%) and TP (64–67%) from simulated wastewater [15]. Furthermore, a laboratory-scale horizontal subsurface flow constructed wetland (HSFCW) planted with *A. calamus* treating anaerobic digester effluent from a swine farm effectively removed COD (17–54%), TP (32–45%), and NH_4_-N (27–88%) at a hydraulic retention time of 54 days and a hydraulic loading rate of 0.01 m^3^ m^−2^ d^−1^ [8].

Even though the application of *A. calamus* for treating wastewater has been studied previously, the knowledge about the growth responses of the plants and its potential for improving the water quality of anaerobic digester effluents is limited. Therefore, this study aims first to determine growth, morphology, pigment contents, and mineral allocation in the plant tissues of *A. calamus* when grown under different concentrations of anaerobic digester effluent (25%, 50%, and 100%) from a swine farm and second to assess the efficiency of *A. calamus* planted systems for water quality improvement. Results from the study will provide useful background information for using *A. calamus* in constructed wetlands treating high-strength anaerobic digester effluent.

## 2. Results

### 2.1. Plant Growth and Morphology

**Figure 1:**
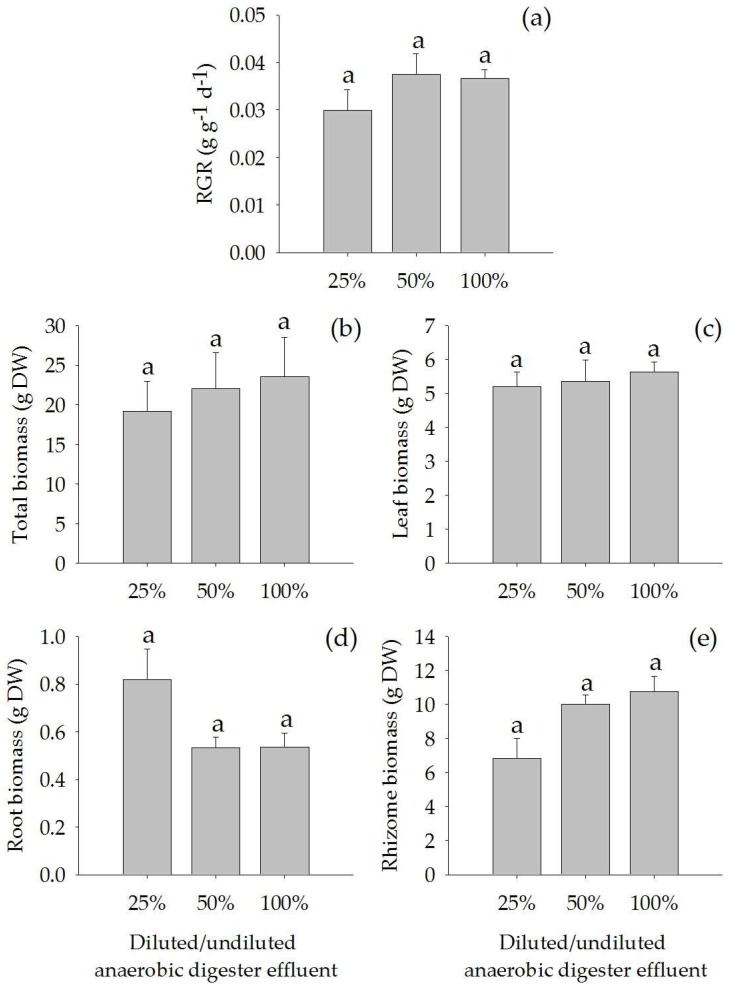
Relative growth rate (RGR) (**a**), total biomass (**b**), leaf biomass (**c**), root biomass (**d**) and rhizome biomass (**e**) (Mean ± SE) of *Acorus calamus* grown on diluted anaerobic digester effluent (25% and 50%) and undiluted anaerobic digester effluent (100%) for a period of 56 days.

**Figure 2:**
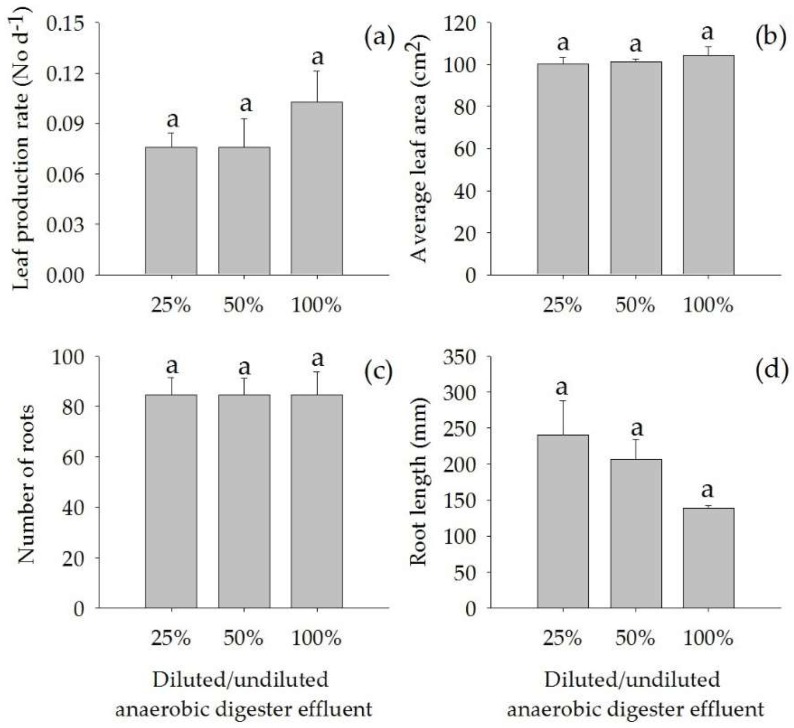
Leaf production rate (**a**), average leaf area (**b**), number of roots (**c**) and root length (**d**) (Mean ± SE) of *Acorus calamus* grown on diluted anaerobic digester effluent (25% and 50%) and undiluted anaerobic digester effluent (100%) for a period of 56 days.

There were no significant differences in relative growth rate, total biomass, leaf biomass, root biomass, rhizome biomass, leaf production rate, average leaf area, number of roots, and root length of *A. calamus* when grown in diluted (25% and 50%) and undiluted (100%) anaerobic digester effluent (Figure 1 and Figure 2).

### 2.2. Pigment Contents

**Figure 3:**
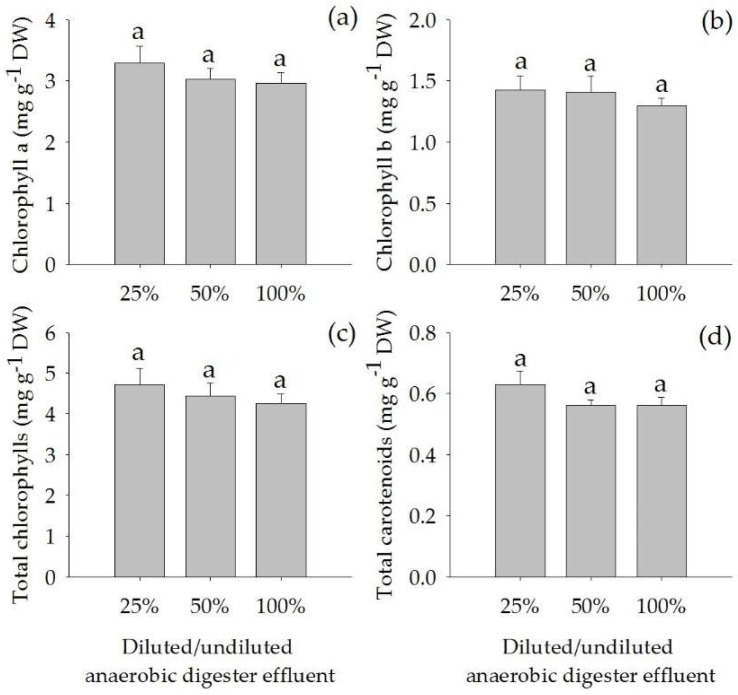
Concentrations of chlorophyll a (**a**), chlorophyll b (**b**), total chlorophylls (**c**) and total carotenoids (**d**) (Mean ± SE) in leaves of *Acorus calamus* grown on diluted anaerobic digester effluent (25% and 50%) and undiluted anaerobic digester effluent (100%) for a period of 56 days.

The concentrations of chlorophyll a, chlorophyll b, total chlorophylls and total carotenoids in the leaves were not significantly different among the treatments (Figure 3). Also, the ratio of Chlorophyll a/b was not significantly different among the treatments (average 2.2 ± 0.0).

### 2.3. NH_4_^+^ and NO_3_^−^ in the Plant Tissue

**Figure 4:**
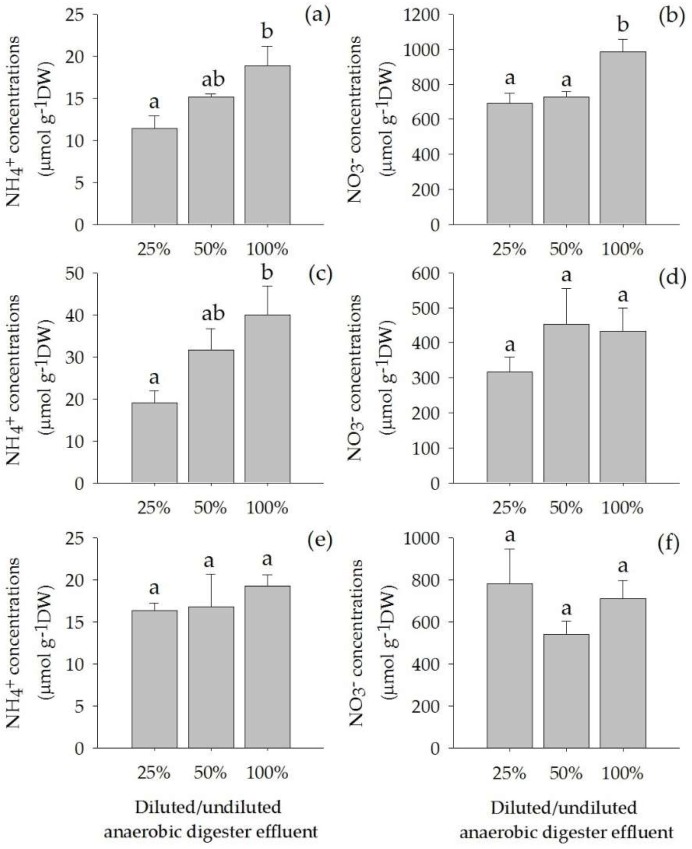
NH_4_^+^ concentration in leaves (**a**), roots (**c**) and rhizomes (**e**) and NO_3_^−^ concentrations in leaves (**b**), roots (**d**) *and* rhizomes (**f**) (Mean ± SE) of *Acorus calamus* grown on diluted anaerobic digester effluent (25% and 50%) and undiluted anaerobic digester effluent (100%) for a period of 56 days. Different letters indicate significant differences among treatments.

The concentrations of anaerobic digester effluent significantly affected the concentrations of NH_4_^+^ and NO_3_^−^ in the leaves and that of NH_4_^+^ in the roots (*p* < 0.05). The NH_4_^+^ concentrations in leaves and roots increased with increasing concentrations of the anaerobic digester effluent (Figure 4a,b). The concentrations of NO_3_^−^ in leaves were highest in plants grown on 100% anaerobic digester effluent (Figure 4b). Nonetheless, the concentrations of NH_4_^+^ in the rhizomes, and NO_3_^−^ in the roots and the rhizomes did not significantly differ among treatments (Figure 4d–f).

### 2.4. Nutrients and Minerals in the Plant Tissue

Both NH_4_^+^ and NO_3_^−^ concentrations in the leaves tended to increase with increasing concentrations of the anaerobic digester effluent, particularly the concentrations of NH_4_^+^ which were high in the roots. Compared with NH_4_^+^ and NO_3_^−^ in the rhizome, there was no significantly difference among treatments. However, NO_3_^−^ concentrations in the plant tissue was much higher than the NH_4_^+^ concentrations (Figure 4).

**Figure 5:**
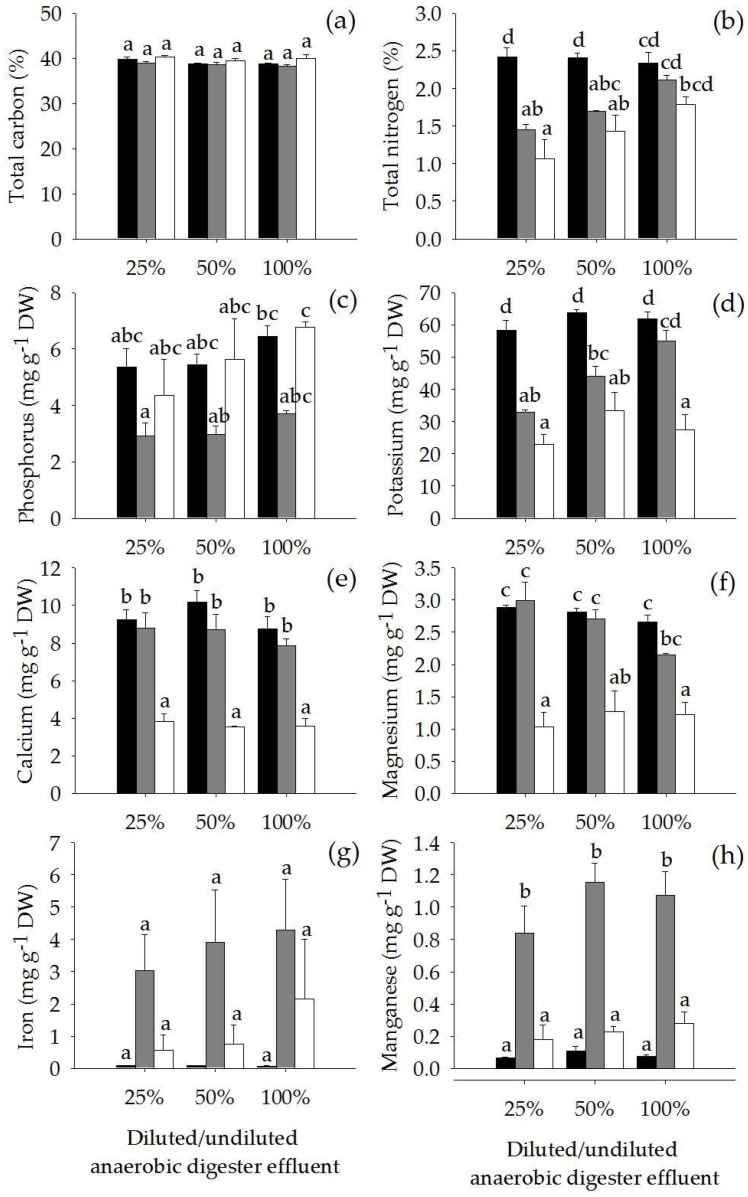
Concentrations of total carbon (**a**), total nitrogen (**b**), phosphorus (**c**), potassium (**d**), calcium (**e**), magnesium (**f**), iron (**g**) and manganese (**h**) (Mean ± SE) in leaves (■), roots (■) and rhizomes (☐) (Mean ± SE) of *Acorus calamus* grown on diluted anaerobic digester effluent (25% and 50%) and undiluted anaerobic digester effluent (100%) for 56 days. Different letters indicate significant differences among treatments.

The concentrations of anaerobic digester effluent significantly affected the concentrations of N, P, K and Mg in the plant tissue (*p* < 0.01). The total N concentrations in the roots and the rhizomes, K in the roots and P in the leaves, increased in the plants treated with high-strength anaerobic digester effluent (Figure 5b–d), whereas, Mg in the roots was reduced (Figure 5f). Moreover, the concentrations of anaerobic digester effluent significantly affected elemental stoichiometry in the plants. The C:N ratios in the roots were significantly lowered (*p* < 0.001) when the plants grew on higher concentrations of anaerobic digester effluent (27.1 ± 1.6, 22.8 ± 0.2 and 18.2 ± 0.5 in 25%, 50% and 100% anaerobic digester effluent, respectively). However, the concentrations of anaerobic digester effluent did not affect total C, Ca, Fe and Mn in the plant tissue (Figure 5a,e,g,h). Similarly, the ratios of C:N in the leaves (average 16.5 ± 0.4) and the rhizomes (average 32.8 ± 4.9), C:P in the leaves, the roots and the rhizomes (average 71 ± 5, 128 ± 10 and 101 ± 27, respectively) and N:P in the leaves, the roots and the rhizomes (average 4.3 ± 0.3, 5.6 ± 0.3 and 2.8 ± 0.2, respectively) were not significantly different among treatments.

### 2.5. Water Quality

**Figure 6:**
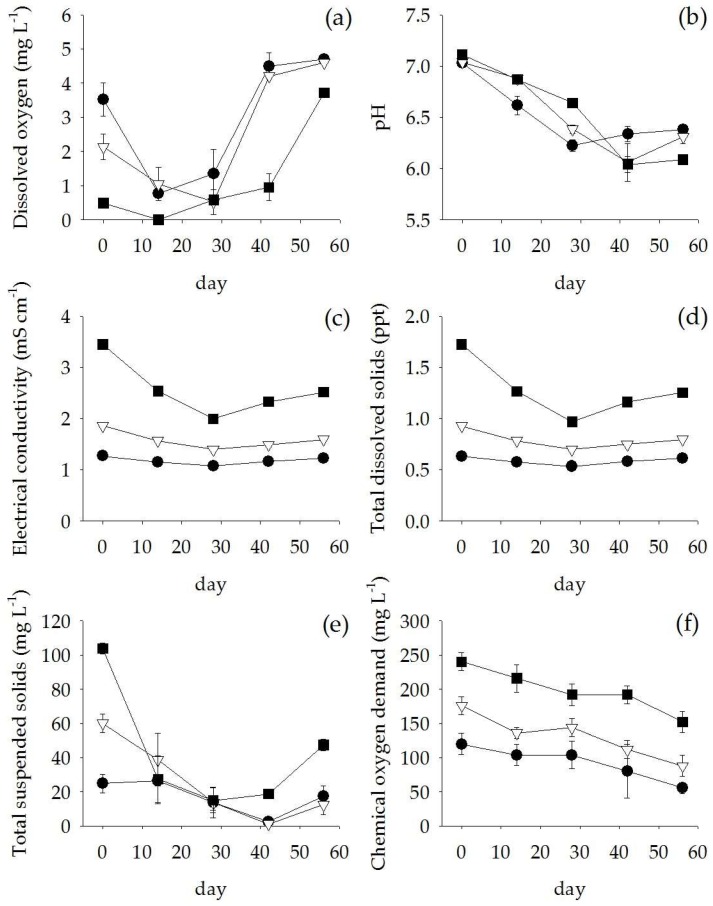
Dissolved oxygen (**a**), pH (**b**), electrical conductivity (**c**), total dissolved solids (**d**), chemical oxygen demand (**e**), and total suspended solids (**f**). (Mean ± SE) in 25% (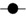) and 50% (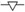) diluted anaerobic digester effluent and 100% anaerobic digester effluent (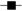) during 56 days of experiment.

**Figure 7:**
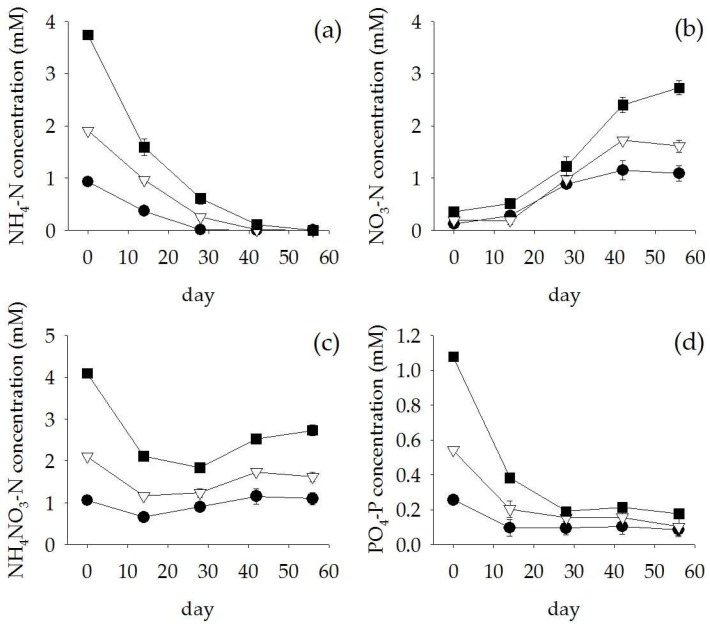
NH_4_-N concentration (**a**), NO_3_-N concentration (**b**), NH_4_NO_3_-N concentration (**c**), and PO_4_-P concentration (**d**) (Mean ± SE) in 25% (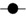) and 50% (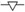) diluted anaerobic digester effluent and 100% anaerobic digester effluent (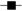) during 56 days of experiment.

After 56 days of treating the anaerobic digester effluent, the water quality generally improved. Dissolved oxygen dramatically decreased during the first two weeks (Figure 6a). The average pH of the anaerobic digester effluent was 6.3 ± 0.2 across the treatments (Figure 6b). Electrical Conductivity (EC) and Total Dissolved Solids (TDS) were slightly reduced (14% and 27% removal) in 50% diluted anaerobic digester effluent and 100% anaerobic digester effluent, respectively (Figure 6c,d). In addition, the TSS in the anaerobic digester effluent was reduced by 40–85% after 28 days and 82–98% after 42 days of treatments (Figure 6e). The COD gradually decreased, reaching a total removal of 37–57% (Figure 6f). For the nutrients, high NH_4_-N removal (>80% removal) was found in all treatments (Figure 7a) whereas the concentrations of NO_3_-N increased to 1.1, 1.6 and 2.7 mM in the 25%, 50%, and 100% anaerobic digester effluent, respectively (Figure 7b). However, the concentration of NH_4_NO_3_-N was reduced by 15–55% within 28 days (Figure 7c). Similarly, PO_4_-P was removed by 64–84% at day 28 for all treatments (Figure 7d).

## 3. Discussion

Overall, *A. calamus* grew well within the effluent from the anaerobic digester treating liquid manure from a swine farm, despite the high nutrient concentrations (undiluted anaerobic digester effluent). The high concentration of the anaerobic digester effluent did not affect pigment contents and minerals in the plant tissue. The nitrogen content was higher in the leaves compared with the roots and rhizomes. Similar results were found in *Phragmites australis* (Cav.) Trin. ex Steud. and *Cyperus malaccensis* var. *brevifolius* Boeckeler, which had higher N concentration in the leaves than the roots and stems [22]. As total C was similar among plant parts and treatments, the leaves had lower C:N ratios than the roots and rhizomes, and the C:N ratio in roots was reduced with increasing concentrations of anaerobic digester effluent. The average C:N ratio of the plants in this study was similar to the average C:N ratios of other aquatic plant species in natural habitats [22,23]. By nature, *A. calamus* prefers high nutrients as it is generally found in nutrients-rich habitats [19]. Likewise, the study of Votjíšková et al. [24] found that *A. calamus* can grow in wastewater with moderately high N (3.75 mM NH_4_-N and 3.75 mM NO_3_-N) and high P (1.5 mM PO_4_-P) concentrations producing high biomass without signs of NH_4_^+^ toxicity. Similarly, the highest concentrations of N and P in the anaerobic digester effluent of this study (NH_4_-N = 3.7 mM, NO_3_-N = 0.4 mM and PO_4_-P= 1.1 mM), which is slightly lower than in the cited previous study, did not affect plant growth. Hence, *A. calamus* can tolerate the high-strength anaerobic digester effluent.

Plants growing in the wastewater treatment systems play an important role to remove excess nutrients from the wastewater and part of nitrogen and phosphorus are removed by root uptake. Many studies have suggested that fast growing species could have high uptake capacity for the nutrients. Hence, most wetland species with fast growth rates have been selected and used for treating the wastewater [25,26,27]. In this study, the relative growth rate of *A. calamus* was 0.01–0.02 g g^−1^ d^−1^ and it was not affected by the strength of the anaerobic digester effluent. Moreover, *A. calamus* tolerated the high nutrient concentrations and did take up significant amounts of N and P from the anaerobic digester effluent. The concentration of inorganic nitrogen in the plant tissue, particularly NO_3_^−^, increased when the plants were exposed to the high strength anaerobic digester effluents. A N mass balance calculation showed that approximately 40% of the nitrogen removed from the undiluted anaerobic digester effluent was accounted for by N assimilation by the plant, resulting in high total N accumulation in the plant tissue. Previous studies have shown that *A. calamus* had high removal efficiency for NH_4_-N (27–88%) and TN (41–63%) when it was used for treating swine digester effluent in horizontal subsurface flow-constructed wetland [8] and treating simulated wastewater in a vertical subsurface flow constructed wetland, respectively [15]. Compared with other studies, the N removal efficiency of *A. calamus* is similar to that of other emergent plants used for treating swine wastewater, such as *T. latifolia*, *Schoenoplectus americanus* (Pers.) Volkart ex Schinz & R. Keller, and *Canna indica* L. [28,29,30].

In the present study, it was found that a high P accumulation in the plant tissues of *A. calamus* was inversely correlated (*p* = 0.026) with the reduction of PO_4_-P in the anaerobic digester effluent. The highest removal of PO_4_-P was found in the undiluted anaerobic digester effluent. After 28 days of treatment, over 80% of PO_4_-P in the anaerobic digester effluent was removed and the concentration was lower than 0.2 mM. Similarly, a study by Fu [31] found that *A. calamus* removed 96.1% of TP when it grew on an artificial solution with 3 mg L^−1^ TP (PO_4_-P, approximately 0.1 mM TP) during 21 days and *A. calamus* had the highest TP removal efficiency compared to other plant species. In CWs, *A. calamus* reduced 64–67% TP from simulated wastewater in a vertical subsurface flow constructed wetland (VSFCW) [15] and 32–45% TP from digested effluent of swine wastewater in a HSFCW [8]. *A. calamus* seems to have a high ability for P removal and a higher removal efficiency for P compared to other emergent plants used in CWs for treating swine wastewater such as *T. latifolia* and *S. americanus* (26–46% TP removal) [28], *T. latifolia* (28% TP removal), *Eleocharis interstincta* (Vahl) Roem. & Schult. (12% TP removal) [29], *C. indica* and *Symphytum officinale* L. (38% TP removal), and *P. australis* (35% TP removal) [30].

Dissolved Oxygen (DO) in the water is one of the most important factors for water quality improvement. The DO in the undiluted anaerobic digester effluent increased significantly during the beginning of the experiment. This could be the consequence of oxygen released from the roots. *Acorus calamus* generally grows in water-logged soils where oxygen is unavailable. Under such conditions, most plants develop air space tissue in the root cortex. Aerenchyma formation is an important response of the plants to provide interconnected air channels that enable gases to diffuse between the plant’s organs. In this study, honeycomb aerenchyma developed in the root cortex of *A. calamus* and oxygen released from the roots, especially from lateral roots (data not shown). Moreover, the plants also had a high number of root and large roots, so root structure could play an important role in increasing DO in the anaerobic digester effluent. Hence, satisfactory removal efficiency of COD was observed, as approximately 37%, 50%, and 53% of the COD were removed from 100%, 50%, and 25% of anaerobic digester effluent, respectively, after 56 days of treatment. However, the COD removal efficiency was slightly lower than those reported from CWs planted with other plant species [8,28,32]. For the TSS removal, a large crowd of roots enhanced precipitation of suspended solids in the anaerobic digester effluent, so TSS dramatically decreased within 28 days, and more than 80% of TSS was removed after 42 days of treatment. This indicates that *A. calamus* is a species that performs well to remove TSS like other species used in constructed wetlands [28,29,32].

## 4. Materials and Methods

### 4.1. Anaerobic Digester Effluent

The wastewater used in the experiment was the effluent from an anaerobic digester used for treating liquid manure from a swine farm with 800 pigs in the Mae Hia campus, Chiang Mai University, Thailand. The characteristics of the anaerobic digester effluent are shown in Table 1.

### 4.2. Plant Culture and Experimental Set-Up

**Figure 8:**
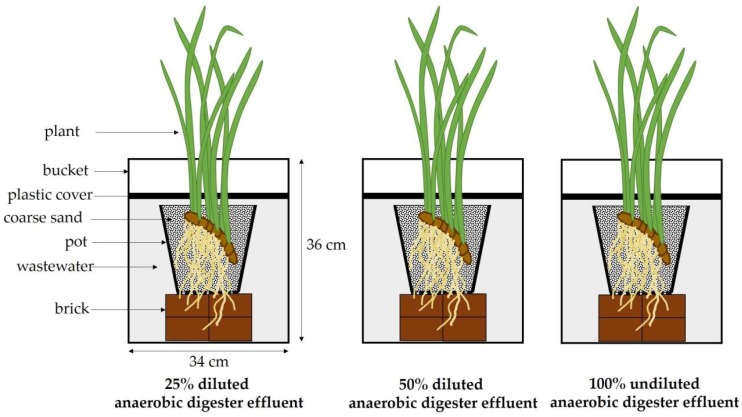
Schematic presentation of the experimental set-up of *Acorus calamus* grown on 25% and 50% diluted anaerobic digester effluent and 100% undiluted anaerobic digester effluent.

The plant stock of *A. calamus* was produced at the greenhouse of the Department of Biology, Faculty of Science, Chiang Mai University. At the beginning, five plants of similar size (approximately 30 g FW and 85 cm high) to the experimental plants were selected, their fresh weight measured and then dried in a ventilated oven at 60 °C to obtain their dry weight. Then, the fresh weight/dry weight ratio (FW/DW ratio) was calculated. For the experiment, twelve plants of similar size (approximately 30 g FW and 85 cm high) were selected. Their initial fresh weight and initial number of leaves were recorded. Then, the plants (*n* = 4) were placed in separate pots (25 cm in diameter and 22 cm in height) with drainage holes containing coarse sand. The plants were fertilized every 2 days with a standard growth medium modified from Smart and Barko [33] with 2 mM N (1 mM NH_4_-N:1 mM NO_3_-N) and 0.1 mM P added. After 7 days, each pot with the individual plant was placed in 15 L plastic buckets (34 cm in diameter and 36 cm in height) containing three different concentrations of the anaerobic digester effluent: 100%, 50% and 25% diluted anaerobic digester effluent, respectively (Figure 8). The water surface was covered with a polyethylene sheet in order to prevent loss of water by evaporation during the experiment. The plants were placed in the greenhouse where the light regimes and temperature were 11 h light/13 h dark and 32 ± 5 °C:26 ± 5 °C day:night, for 56 days.

### 4.3. Plant Growth and Morphology

At day 56, all plants were harvested. The number of leaves, average leaf area, number of roots and root length were recorded. The leaf production rate (No d^−1^) was calculated as the increase in number of leaves throughout the experiment divided by the number of experiment days. Then, the plants were fractionated into leaves, roots and rhizomes. Half of the plant samples were dried in a freeze dryer (Alpha 1-4 LD plus, Martin Christ, Osterode, Germany) and the other plant samples were dried in a ventilated oven at 60 °C until constant weight. The leaf, root and rhizome biomass was recorded and the Relative Growth Rate (RGR, g g^−1^ d^−1^) was calculated by using the formula: RGR = (lnW_2_ − lnW_1_)/(t_2_ − t_1_), where W_1_ and W_2_ are the initial and final total dry weight (g) and t_1_ and t_2_ are the initial and final time (days).

### 4.4. Pigments

Photosynthetic pigments (chlorophylls and carotenoids) in the leaves were analyzed in samples (8 mg) of finely-ground freeze-dried leaves. The samples were extracted in 96% ethanol (8 mL) in a dark room at room temperature for 24 h. Then the absorbance of the extracts was measured at 470 nm, 648.6 nm and 664.2 nm using a UV-VIS Spectrophotometer (UV-1800, Shimadzu Co., Kyoto, Japan). The concentrations of chlorophyll a, chlorophyll b, total chlorophylls and total carotenoids were calculated according to Lichtenthaler [34].

### 4.5. Nutrients and Minerals in Plant Tissue

The NH_4_^+^ and NO_3_^−^ in the leaves, roots and rhizomes were extracted using a hot water extraction method. Approximate 5 mg of finely-cut freeze-dried leaves, roots and rhizomes were placed in 15 mL of distilled water in screw-cap centrifuge tubes. Then, the sample tubes were incubated in a water bath at 80 °C for exactly 20 min. After that, 2.5 mL of the sample solutions were withdrawn, diluted with 2.5 distilled water and analyzed for NH_4_^+^ concentration using a modified salicylate method (Quikchem Method No. 10-107-06-3-B; Lachat Instruments, Milwaukee, WI, USA). Two and a half milliliters of the sample solutions were withdrawn, diluted with 2.5 distilled water and analyzed for NO_3_^−^ concentration using an ultraviolet (UV) method [35].

Total carbon (C) and nitrogen (N) concentrations in the leaves, roots and rhizomes were analyzed. Approximately 2 mg of finely-ground freeze-dried plant materials were analyzed using a CHNS analyzer (vario EL cube Elementar, Elementar Analysensysteme GmbH, Hanau, Germany). The concentrations of potassium (K), phosphorus (P), calcium (Ca), magnesium (Mg), iron (Fe), and manganese (Mn) in the leaves, roots, and rhizomes were analyzed from finely-ground freeze-dried plant materials. Approximately 200 mg of subsamples were digested with 4 mL of 65% HNO_3_ and 2 mL of H_2_O_2_ in a microwave sample preparation system (Multiwave 3000, Anton Paar GmbH, Austria) and the elemental concentrations were analyzed using inductively coupled plasmaspectrometry (Optima 2000 DV, PerkinElmer Instruments Inc., Shelton, CT, USA).

### 4.6. Water Analysis

The water samples (800 mL) from each treatment were collected at the beginning of the experiment and every 14 days after experimental set-up for a total period of 56 days. Electrical Conductivity (EC), Total Dissolved Solids (TDS), and pH were measured using a multi-parameter analyzer (CyberScan PC 300, Eutech Instruments Pte Ltd., Singapore). Dissolved Oxygen (DO) was analyzed by an azide modification method (4500-O C) [36]. Chemical Oxygen Demand (COD) was analyzed by a close reflux, titrimetric method (5220 C) [36]. Total Suspended Solids (TSS) was analyzed using a standard method 2540 D [36]. Then, the filtered water samples were analyzed for concentrations of ammonium nitrogen (NH_4_-N), nitrate nitrogen (NO_3_-N) and orthophosphate (PO_4_-P). Ammonium nitrogen concentration was analyzed using a modified salicylate method (Quikchem Method no. 10-107-06-3-B; Lachat Instruments, Milwaukee, WI, USA). Nitrate nitrogen concentration was analyzed by an UV-method [35]. Orthophosphate was analyzed by a stannous chloride method (4500-P D) [36].

### 4.7. Statistics

All data were statistically analyzed using SPSS Statistics version 17.0 (SPSS Inc., Chicago, IL, USA) [37]. The normality and homogeneity of variance were tested by using the Shapiro-Wilk test and Levene Statistic, respectively. If necessary, logarithmic or square root transformations were performed to ensure homogeneity of variance. Then, the data with normal distribution were tested by one-way analysis of variance (ANOVA) and the differences between treatments were identified using the Tukey HSD post hoc procedure at the 5% significance level. The data with non-normal distribution were tested by the Kruskal-Wallis test at 5% significance level.

## 5. Conclusions

In conclusion, the indifferences of growth, morphology and pigment contents of *A. calamus* under different concentrations of anaerobic digester effluent suggests that this species has the ability to grow and tolerate the high nutrient concentrations in the effluent from anaerobic digesters treating manure from swine farms. This plant also had elevated concentrations of NH_4_^+^, NO_3_^−^, TN, and TP in the plant tissues when the plant grew on higher concentrations of the effluent. Furthermore, *A. calamus* has high efficiencies for removing orthophosphate and inorganic nitrogen, as well as high efficiency for removing COD from the anaerobic digester effluent. Therefore, *A. calamus* could be suggested as an effective species for treating wastewater, particularly wastewater containing high N and P concentrations, such as effluents from anaerobic digesters treating wastes from animal farms as well as other types of agricultural wastewater.

## Figures and Tables

**Table 1:** Characteristics of anaerobic digester effluent from swine farm.

Parameters	Mean values
Water temperature (°C)	29.1
pH	7.1
Electrical conductivity (mS cm^−1^)	3.4
Dissolved oxygen (mg L^−1^)	0.5
Total dissolved solids (ppt)	1.7
Total suspended solids (mg L^−1^)	103.8
Chemical oxygen demand (mg L^−1^)	192.0
NH_4_-N concentration (mM)	3.7
NO_3_-N concentration (mM)	0.4
Orthophosphate (PO_4_-P) concentration (mM)	1.1

## References

[1] Hairston J.E., Stribling L. (1995). Animal Waste Management to Protect Water Quality: Animal Waste and How it Affects Water Quality.

[2] World Health Organization (2012). Animal Waste, Water Quality and Human Health.

[3] Bruinsma J. (2003). World Agriculture: Towards 2015/2030—An FAO Perspective.

[4] Chen Y., Cheng J.J., Creamer K.S. (2008). Inhibition of anaerobic digestion process: A review. Bioresour. Technol..

[5] Khalid A., Arshad M., Anjum M., Mahmood T., Dawson L. (2011). The anaerobic digestion of solid organic waste. Waste Manag..

[6] Lee C.Y., Lee C.C., Lee F.Y., Tseng S.K., Liao C.J. (2004). Performance of subsurface flow constructed wetland taking pretreated swine effluent under heavy loads. Bioresour. Technol..

[7] Deng L., Zheng P., Chen Z., Mahmood Q. (2008). Improvement in post-treatment of digested swine wastewater. Bioresour. Technol..

[8] Liu G.J., Zheng D., Deng L.W., Wen Q., Liu Y. (2014). Comparison of constructed wetland and stabilization pond for the treatment of digested effluent of swine wastewater. Environ. Technol..

[9] Wen S., Liu H., He H., Luo L., Li X., Zeng G., Zhou Z., Lou W., Yang C. (2016). Treatment of anaerobically digested swine wastewater by *Rhodobacter blasticus* and *Rhodobacter capsulatus*. Bioresour. Technol..

[10] Kayhanian M. (1999). Ammonium inhibition in high-solids biogasification: An overview and practical solutions. Environ. Technol..

[11] Sánchez E., Borja R., Weiland P., Travieso L., Martín A. (2000). Effect of temperature and pH on the kinetics of methane production, organic nitrogen and phosphorus removal in the batch anaerobic digestion process of cattle manure. Bioprocess Eng..

[12] Vymazal J. (2010). Constructed wetlands for wastewater treatement. Water.

[13] Brix H. (1997). Do macrophytes play a role in constructed treatment wetlands?. Water Sci. Technol..

[14] Karathanasis A.D., Potter C.L., Coyne M.S. (2003). Vegetation effects on fecal bacteria, BOD and suspended solid removal in constructed wetlands treating domestic wastewater. Ecol. Eng..

[15] Zhao Y., Liu B., Zhang W., Kong W., Hu C., An S. (2009). Comparison of the treatment performances of high-strength wastewater in vertical subsurface flow constructed wetlands planted with *Acorus calamus* and *Lythrum salicaria*. J. Health Sci..

[16] Motley T.J. (1994). The ethnobotany of sweet flag, *Acorus calamus* (Aracaceae). Econ. Bot..

[17] Bhat S.D., Ashok B.K., Bhat D.V., Acharya R., Shukla V.J. (2011). A comparative phytochemical evaluation of wild and cultivated *Acorus calamus* Linn (vacha) with special reference to β-Asarone content. Inven. Rapid Pharm Anal. Qual. Assur..

[18] Singh R., Sharma P.K., Malviya R. (2011). Pharmacological properties and Ayurvedic value of Indian buch plant (*Acorus calamus*): A short review. Adv. Biol. Res..

[19] Dykyjová D. (1980). Production ecology of *Acorus calamus*. Folia Geobot. Phytotxon..

[20] Weber M., Brändle R. (1994). Dynamics of nitrogen-rich compounds in roots, rhizomes and leaves of the sweet flag (*Acorus calamus* L.) at its natural site. Flora.

[21] Weber M., Brändle R. (1996). Some aspects of the extreme anoxia tolerance of the sweet flag, *Acorus calamus* L.. Folia Geobot. Phytotxon..

[22] Wang W.Q., Sardans J., Wang C., Zeng C.S., Tong C., Asensio D., Penuelas J. (2015). Ecological stoichiometry of C, N and P of invasive *Phragmites australis* and native *Cyperus malaccensis* species in the Minjiang River tidal estuarine wetlands of China. Plant Ecol..

[23] Frost P.C., Hicks A.L. (2012). Human shoreline development and the nutrient stoichiometry of aquatic plant communities in Canadian Shield lakes. Can. J. Fish. Aquat. Sci..

[24] Votjíšková L., Munzarová E., Votrubová O., Řihová A., Juřicová B. (2004). Growth and biomass allocation of sweet flag (*Acorus calamus* L.) under different nutrient conditions. Hydrobiologia.

[25] Tanner C.C. (1996). Plants for constructed wetland treatment systems—A comparison of the growth and nutrient uptake of eight emergent species. Ecol. Eng..

[26] Calheiros C.S.C., Quitério P.V.B., Silva G., Crispim L.F.C., Brix H., Moura S.C., Castro P.M.L. (2012). Use of constructed wetland systems with *Arundo* and *Sarcocornia* for polishing high salinity tannery wastewater. J. Environ. Manag..

[27] Vincent G., Shang K., Zhang G., Chazarenc F., Brisson J. (2017). Plant growth and nutrient uptake in treatment wetlands for water with low pollutant concentration. Water Sci. Technol..

[28] Poach M.E., Hunt P.G., Reddy G.B., Stone K.C., Johnson M.H., Grubbs A. (2007). Effect of intermittent drainage on swine wastewater treatment by marsh–pond–marsh constructed wetlands. Ecol. Eng..

[29] González F.T., Vallejos G.G., Silveira J.H., Franco C.Q., García J., Puigagut J. (2009). Treatment of swine wastewater with subsurface-flow constructed wetlands in Yucatán, Mexico: Influence of plant species and contact time. Water SA.

[30] Borin M., Politeo M., Stefani G.D. (2013). Performance of a hybrid constructed wetland treating piggery wastewater. Ecol. Eng..

[31] Fu X., Yarlagadda P. (2015). Phosphorus removal from wastewater by five aquatic plants. Proceedings of the 3rd International Conference on Advances in Energy and Environmental Science 2015.

[32] Klomjek P. (2016). Swine wastewater treatment using vertical subsurface flow constructed wetland planted with Napier grass. Sustain. Environ. Res..

[33] Smart R.M., Barko J.W. (1985). Laboratory culture of submersed freshwater macrophytes on natural sediments. Aquat. Bot..

[34] Lichtenthaler H.K. (1987). Cholophylls and carotenoids: Pigments of photosynthetic biomembranes. Methods Enzymol..

[35] Cedergreen N., Madsen T.V. (2002). Nitrogen uptake by the floating macrophyte *Lemna minor*. New Phytol..

[36] American Public Health Association, American Water Works Association, Water Environment Federation (1998). Standard Methods for the Examination of Water and Wastewater.

[37] SPSS (2007). SPSS for Windows, Standard Version 17.0.

